# Shelf Life Extension and Quality Improvement of Fresh Ostrich Meat by Active Chitosan Coating Enriched With Fennel Extract

**DOI:** 10.1002/fsn3.70262

**Published:** 2025-05-09

**Authors:** Mahdieh Salari, Motahareh Yazdany

**Affiliations:** ^1^ Department of Food Science and Technology, Faculty of Agriculture University of Tabriz Tabriz Iran; ^2^ Department of Food Science and Technology, Faculty of Technical and Engineering University of Science and Arts of Yazd Yazd Iran

**Keywords:** chitosan coating, fennel extract, ostrich meat, shelf life extension

## Abstract

The present study aimed to evaluate the effect of chitosan (CS, 2%, w/v), fennel extract (FE, 1.5%, v/v), and CS + FE (2%, w/v CS + 1.5%, v/v FE) coatings on the physicochemical, microbial, and sensory properties of ostrich meat (OM) at 0, 7, and 15 days of refrigerated storage. The obtained results showed that the use of coatings for OM samples demonstrated a significant enhancement in moisture retention and a decrease in pH, thiobarbituric acid reactive substances (TBARS), total volatile nitrogen (TVB‐N), and peroxide value (PV) values compared to the control one, which were 6.34, 0.53 mg/kg, 15.14 mg/100 g, and 2.64 meq/kg, respectively for the CS + FE coating sample, at the 15th day. At the end of the storage time, the total viable count (TVC), psychrophilic bacteria count (PBC), and lactic acid bacteria count (LABC) of the control sample notably (*p* < 0.05) decreased from 8.92, 6.86, and 5.45 to 6.24, 5.78, and 3.2 log10 CFU/g when coated with CS + FE, respectively. Additionally, sensory evaluation revealed that the treatments did not remarkably affect the initial sensory characteristics of OM and decreased their deterioration throughout storage. In conclusion, the application of the CS + FE coating could be considered a promising alternative to artificial preservatives for meat and meat products, as it extends the shelf life of OM in the refrigerator by more than 2 weeks.

## Graphical abstract

FE, as a natural preservative, was added to the CS solution for active coating preparation. The CS + FE coating limited the TBARS, TVB‐N, and PV increase in ostrich meat. FE‐enriched CS coating demonstrated antioxidant and antimicrobial impacts on ostrich meat. CS + FE coating could be considered an effective method to extend the shelf life of fresh ostrich meat.

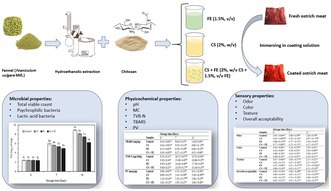

## 1 Introduction

Ostrich meat is considered a healthier alternative to traditional red meats because it contains less cholesterol and fat while offering three times more omega‐3 fatty acids. However, factors like packaging, storage conditions (such as light, temperature, humidity), initial microbial load, and pH levels can significantly impact the quality of ostrich meat (Heydari et al. 2020). For example, the elevated pH level of ostrich meat (≅ 6) is a factor that can contribute to rapid microbial spoilage in packaged samples (Divani et al. 2018). Spoilage microorganisms multiply rapidly in meat, speeding up the degradation of lipids and proteins, which increases rancidity. This not only reduces nutritional value but also negatively impacts the meat's sensory qualities such as color, texture, and odor ultimately lowering its overall quality and shelf life (Alizadeh Behbahani et al. 2020). Extending the shelf life of fresh raw meat remains a key challenge in the meat industry. Conventional chemical preservatives such as sodium nitrate, potassium sorbate, and benzoic acid have been associated with negative health effects. Because of this, researchers are increasingly exploring natural alternatives, including essential oils and extracts derived from plants (Kiarsi et al. 2020; Smeti et al. 2025), bacteriocins (Cocolin 2025), modified atmosphere packaging (Rothy et al. 2025), and organic acids (Tellini et al. 2024) to extend the shelf life and inhibit lipid oxidation in raw meat. Additionally, due to rising consumer demand for high‐quality food and growing concerns over plastic waste, researchers are now prioritizing biodegradable films and edible coatings. These coatings apply a thin protective layer to food surfaces, including meat, vegetables, and fruits. This helps maintain postharvest or postslaughter quality, thereby extending shelf life. Among current methods, edible and biodegradable coatings are particularly promising, as they effectively preserve food while offering environmental benefits (Behbahani and Imani Fooladi 2018). In addition, these coatings can carry food additives such as antimicrobial compounds, antioxidants, and vitamins, effectively functioning as active packaging (Alizadeh Behbahani et al. 2017; de Lima et al. 2024). Because of these benefits, edible coatings and films have gained more acceptance from consumers compared to other preservation methods like irradiation and the use of chemical preservatives (Saha et al. 2017).

Chitin, the second most abundant polysaccharide in nature after cellulose (Mehdizadeh et al. 2022), is commonly found in the shells of crustaceans like shrimp and crabs, as well as in the cell walls of fungi and the exoskeletons of insects. Chitosan is a linear, nontoxic, biocompatible, and biodegradable polysaccharide derived from chitin through deacetylation (Mahin et al. 2025). Chitosan, being an antimicrobial and antioxidant material, is generally recognized as safe (GRAS), making it suitable for various food‐related applications. It can be effectively utilized to produce edible coatings and films for food packaging purposes (Mojaddar Langroodi et al. 2021).

Active coatings are a highly effective way to improve food safety. They slowly release active ingredients such as plant extracts and essential oils (EOs) without directly mixing them into the food. This controlled release helps maintain quality and extend the shelf life of the final product (Jridi et al. 2018). Fennel ( *Foeniculum vulgare* Mill.), belonging to the Apiaceae family, holds significant importance as a medicinal plant (Song et al. 2024). Fennel is a resilient perennial plant known for its ability to stimulate appetite and enhance digestion. Additionally, it has been traditionally used to address various health concerns, including menopausal symptoms, kidney stones, obesity, and nausea (Ehsanipour et al. 2012). Fennel seeds are rich in potent natural antioxidants, including vitamins C and E, phenolic compounds, and oleoresins. These antioxidants exhibit strong antioxidant properties in both foods and biological systems (Oktay et al. 2003). The volatile components found in fennel seed extract encompass a variety of compounds such as α‐pinene, trans‐anethole, limonene, terpenes, estragole, geraniolene, methyl chavicol, α‐ferranene, and fenchone. These components collectively contribute to an effective bactericidal effect (Mohamad et al. 2011; Sun et al. 2021). Liu et al. (2020) utilized a fennel essential oil nanoemulsion with polylysine to preserve the quality of ready‐to‐eat Yao meat. Their findings revealed that the developed coating exhibited significant antibacterial properties. In the study conducted by Diao et al. (2014), the chemical composition of fennel essential oil was thoroughly investigated, revealing its antibacterial properties and elucidating its antibacterial mechanism against various pathogens including *Escherichia coli* , *Bacillus subtilis* , *Staphylococcus albus* , *Shigella dysentery*, and *Salmonella typhimurium* .

Thus far, chitosan films and coatings integrated with extracts and essential oils (EOs) have been effectively employed to maintain the quality of a range of products, including seafood (Morachis‐Valdez et al. 2021), meat (Isvand et al. 2024) and meat products (Ghaderi et al. 2024), as well as vegetables (Saren et al. 2022) and fruits (Phuong et al. 2024). Homayonpour et al. (2021) exhibited an extension of sardine shelf life by measuring microbial and chemical tests in sardine fillets kept by *Cumino cyminum* L. essential oil (CCEO) encapsulated nanochitosan coatings. In another study, Mokarami et al. (2022) studied the effects of nanochitosan–zein coating containing *Mentha pulegium* L. extract on the shelf life of Persian shrimp (*Fenneropenaeus persian*). The authors stated that the use of this coating resulted in delayed chemical and microbial spoilage and increased shrimp shelf life during cold storage.

Considering the increasing consumption of ostrich meat in recent years and, on the other hand, as far as we know, there is no report on the use of chitosan coating enriched with fennel extract for the preservation of ostrich meat, the present research seems necessary and important. Consequently, this study, for the first time, was undertaken to assess the impact of CS coating containing FE on the microbial and physicochemical characteristics of ostrich meat stored at 4°C for a duration of 15 days. Additionally, the study aimed to explore the influence of this treatment on the sensory attributes of both coated and uncoated samples over the storage period.

## 2 Materials and Methods

### 2.1 Materials

Commercial shrimp skin chitosan powder (medium molecular weight (270 kDa), deacetylation degree of 85%) was obtained from Sigma Aldrich Chemical Co., Germany. Glacial acetic acid, pH buffer 4 and 7, thiobarbituric acid (TBA), ethanol (96% v/v), magnesium oxide (MgO), trichloroacetic acid (TCA), boric acid, methyl red, methylene blue, sulfuric acid, chloroform, potassium iodide, sodium thiosulfate, peptone water, plate count agar (PCA), and Mann Rogosa Sharpe (MRS) agar were supplied by Merck Co., (Germany). Analytical‐grade chemicals were used for all tests.

### 2.2 Measuring Devices Specifications

Rotary evaporator (Heidolph Instruments Co., VV 2000, Germany), homogenizer (Heidolph Silent Crusher M, Germany), pH meter (STARTER 3000, OHAUS Co., Switzerland), oven (Shimaz Co., Iran), centrifuge (Hettich Universal 320, Sigma Co., Germany), spectrophotometer (Pharmacia Biotech Co., England), Kjeldahl apparatus (Kjeltec PDU 500, PECO, Iran), and stomacher (BagMixer 400, Interscience Co., France) were used.

### 2.3 Preparation of Fennel Extract

For extraction, the hydroethanolic extraction method was performed according to the report of Mojaddar Langroodi et al. (2018) with some modifications. To begin, 50 g of dry fennel seeds was obtained from a local shop (Yazd, Iran) and then ground into a fine powder by an electric mill. This powder was then combined with 700 mL of ethanol and 300 mL of distilled water in a decanter. The mixture was allowed to undergo extraction for a period of 24 h. After extraction, the obtained extracts were filtered through Whatman 40 filter paper to remove any solid particles or impurities. Subsequently, a rotary evaporator was employed under vacuum conditions at 45°C to concentrate the extracts. Finally, the concentrated extracts were stored at 4°C until they were ready for use in further experiments.

### 2.4 Preparation of Coating Solutions

To prepare the chitosan solutions (2% w/v), chitosan powder was mixed with 1 L of distilled water. The mixture was stirred for 10 min at 60°C, and then 10 mL of glacial acetic acid (1% v/v) was added to it, and stirring was continued for an additional 60 min. This step ensured proper dispersion and dissolution of the chitosan powder in the solution (Kanatt et al. 2013). To prepare the active chitosan coating, fennel extract was dissolved in the chitosan solution with a concentration of 1.5% (v/v). The final active coating solution was homogenized at 22,000 rpm for 60 s under aseptic conditions.

To determine the optimal concentration of fennel extract in a 2% chitosan solution, a preliminary study was conducted. In this regard, concentrations of 0.3%, 0.6%, 0.9%, 1.5%, and 2.0% (v/v) of fennel extract were prepared in chitosan solution. The obtained results showed that the concentration of fennel extract < 1.5% v/v was not effective enough for the intended purposes, while the concentration > 1.5% had a negative effect on the sensory characteristics of the samples. Therefore, fennel extract with a concentration of 1.5% was used in this study.

### 2.5 Preparation of Meat Samples

A sufficient quantity of fresh ostrich meat (22.95% ± 0.23% protein, 75.61% ± 0.14% moisture, 1.15% ± 0.07% fat, and 1.42% ± 0.02% ash) was obtained from a local butcher shop in Yazd, Iran, on the day of slaughtering. The ostrich meat was deboned and skinned using an aseptic technique, then cut uniformly into portions (100 g, 2.5 cm thick) with a sterile surgical knife. The meat samples were then individually immersed in a freshly prepared coating solution for 3 min. Subsequently, they were placed under a biological hood for 2 h at 10°C to allow the formation of edible coatings and then stored at 4°C for 15 days. For all samples, a ratio of 2:1 ostrich meat to solution (weight/volume) was maintained. The samples were randomly divided into four different treatment groups: control (uncoated), CS, 1.5% FE, and CS + 1.5% FE. The quality of the ostrich meat was assessed periodically at intervals of 0, 7, and 15 days during the storage period. Each experimental treatment was conducted in triplicate to ensure the accuracy and reliability of the results.

### 2.6 pH Determination

For pH determination, a pH meter was initially calibrated using standard phosphate buffers of pH 4 and pH 7. Following calibration, 5 g of each sample was homogenized in 20 mL of distilled water using a homogenizer set at 9500 rpm for 1 min. The resulting mixture was allowed to settle for 10 min. pH measurements were then conducted at a temperature of 25°C ± 1°C (Vidal et al. 2024).

### 2.7 Moisture Content (MC) Determination

The moisture content of the ostrich meat samples was determined by drying them in an oven at 110°C for 24 h, with the initial (W_i_, g) and final (W_f_, g) constant weights used for the calculation (Phuong et al. 2024):

(1) $$\mathrm{MC}\;\left(\%\right)=\frac{W_{i}-W_{f}}{W_{i}}\times 100$$

### 2.8 Thiobarbituric Acid Reactive Substances (TBARS) Determination

To evaluate the value of TBARS, the Jridi et al. (2018) method was used with some changes. In brief, 5 g of meat samples was mixed with 20 mL of diluted trichloroacetic acid (TCA) up to 5% and homogenized using a homogenizer for 10 min. The homogenate was then subjected to centrifugation at 12,000*g* for 15 min at 4°C. Following centrifugation, 4 mL of the supernatant was transferred into a test tube, and 4 mL of 0.02 M thiobarbituric acid (TBA) was added. The mixture was then stirred using a vortex and placed in a hot water bath at 100°C for 60 min. After incubation, the tubes were immediately cooled under running water for 10 min. Subsequently, the resulting mixture was centrifuged at 3000*g* for 8 min at 4°C. The absorbance of the supernatant was measured using a spectrophotometer at 532 nm. The TBARS value was calculated based on the concentration of malonaldehyde, expressed as milligrams (mg) of malonaldehyde per kilogram (kg) of the sample.

### 2.9 Total Volatile Nitrogen (TVB‐N) Determination

For total volatile basic nitrogen (TVB‐N) determination, homogenized samples were mixed with magnesium oxide (MgO) and distilled using a Kjeldahl apparatus. The distillate was collected in an aqueous solution of boric acid (3%) containing a mixed indicator obtained by dissolving methyl red (0.1 g) and methylene blue (0.1 g) in ethanol (100 mL). The boric acid solution was then titrated with a sulfuric acid solution (0.05 M). The TVB‐N value was calculated based on the amount of sulfuric acid consumed during titration and expressed as milligrams (mg) of nitrogen per 100 g (g) of ostrich meat (Ojagh et al. 2010).

### 2.10 Peroxide Value (PV) Determination

PV is evaluated to determine primary lipid oxidation products. For peroxide index evaluation, 3 g of lipid was extracted from the samples according to the Bligh and Dyer (1959) procedure and then combined with 30 mL of glacial acetic acid solution and chloroform in a mixing ratio of 3:2, respectively, along with 0.5 mL of potassium iodide saturated solution. The resulting mixture was allowed to stand in a dark room for 1 min. Next, 30 mL of distilled water and 0.5 mL of starch suspension (1% concentration, used as an indicator) were added to the mixture. The final solution was titrated with sodium thiosulfate (0.01 N) until the color disappeared, indicating the completion of the reaction. The PV (meq O_2_/kg lipid) was reported based on the following equation (Sahraee et al. 2020):

(2) $$\mathrm{PV}=\frac{100\times V\times N}{W}$$

where V, N, and W are sodium thiosulfate volume, sodium thiosulfate normality, and lipid weight, respectively.

### 2.11 Microbiological Analysis

The microbiological quality determination of ostrich meat samples involved determining three key indicators of microbial spoilage: total viable counts (TVC), psychotropic bacteria counts (PBC), and lactic acid bacteria counts (LABC). Approximately 5 g of each sample was homogenized with 45 mL of 0.1% peptone water solution using a stomacher for 1 min under aseptic conditions to prepare serial dilutions (1:10). The pour plate method was then employed on preprepared agar plates containing plate count agar (PCA) medium for TVC and PBC enumeration, with incubation at 37°C for 48 h and at 7°C for 10 days, respectively. This enabled the measurement of microbial levels associated with spoilage (Faradonbeh et al. 2024). For the measurement of lactic acid bacteria (LAB) growth, the same sample preparation method was followed, and serial dilutions were prepared. Subsequently, 1 mL of each dilution was inoculated onto Mann Rogosa Sharpe (MRS) agar medium using the standard plate count method. The inoculated plates were then placed in an incubator set at a temperature of 37°C for a duration of 48 h. This incubation period allowed for the growth and enumeration of lactic acid bacteria present in the samples (Lashkari et al. 2020). The results of bacterial enumerations were converted to log_10_ CFU/g.

### 2.12 Determination of Sensory Properties

Twelve trained panelists (six females and six males, age: 20–39 years) were enlisted to evaluate the sensory attributes of ostrich meat (OM) samples, focusing on odor, color, texture, and overall acceptability. Before the test, all panelists were trained in techniques for assessing the sensory attributes and spoilage indicators of fresh ostrich meat. Both treated and control samples were assigned random three‐digit codes and presented to the participants in clean stainless‐steel trays at room temperature. The evaluation was conducted using the hedonic scoring system, where a score of 1 indicated “extremely unacceptable” and a score of 5 indicated “extremely acceptable.” Ostrich meat samples receiving an overall score of more than 4 were deemed acceptable based on sensory evaluation (Majdinasab et al. 2020).

### 2.13 Statistical Analysis

For data analysis, a completely randomized design was employed with three replications. Means were compared using a one‐way analysis of variance (ANOVA) followed by Duncan's multirange test at a significance level of 5%. Statistical analysis was conducted using SPSS 25 software (SPSS Inc., Chicago, USA), and graphs were generated using Excel software.

## 3 Results and Discussion

### 3.1 pH

**FIGURE 1:**
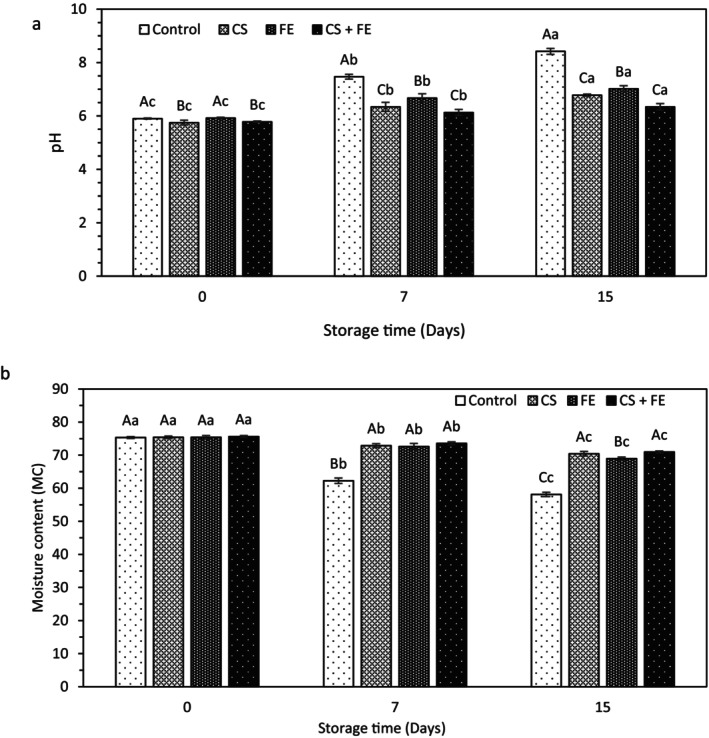
Changes in pH (a) and moisture content (MC) (b) of the ostrich meat samples during refrigerated storage (4°C) for 15 days. Different capital letters indicate significant differences among treatments, and different small letters indicate significant differences in storage days (*p* < 0.05).

One of the key quality indicators for fresh meat is pH. As outlined in Figure 1a, the initial pH value of the treated ostrich meat (OM) samples was insignificantly lower than that of the control sample (*p* > 0.05), likely due to the presence of acetic acid in the chitosan solution used for treatment. The pH values of the uncoated control sample steadily and significantly increased from 5.9 on Day 0 to 8.42 on Day 15 (*p* < 0.05). These findings are in line with previous observations indicating a rise in pH values of uncoated raw meat over the course of cold storage (Behbahani et al. 2017; Mojaddar Langroodi et al. 2018; Noshad et al. 2021). The observed increase in pH value is attributed to meat spoilage, which involves the production of nitrogenous basic compounds such as ammonia and amines. These compounds are generated due to the breakdown of proteins by proteolytic enzymes that are secreted by microorganisms during spoilage (Triki et al. 2018). Indeed, the rise in pH can adversely impact product quality during storage, particularly affecting sensory attributes such as texture, color, and odor (Zhang et al. 2016).

From the 7th day onwards, there was a notably greater increase in pH value observed in the control sample compared to the other samples (*p* < 0.05). The CS‐coated samples had a lower pH than the samples of ostrich meat coated with FE. The observed pH changes in stored ostrich meat may be attributed to chitosan's strong antimicrobial and antioxidant properties, which inhibit microbial growth and prevent proteolysis. However, on the last day of the storage period (Day 15), the pH of the samples treated with chitosan (CS) or fennel extract (FE) was higher than that of the CS + FE coated sample, which can indicate the significant effect of the simultaneous use of chitosan coating and fennel extract in reducing microbial spoilage (*p* < 0.05). These findings are consistent with previous research (Jridi et al. 2018; Kamkar et al. 2021), which found that meat samples coated with active coating exhibited the lowest pH value compared to other samples. It is described in the studies of Zomorodian et al. (2023) and Zheng et al. (2023) that chitosan enriched with plant essential oils has been demonstrated to decrease the pH values of salmon ( *Salmo trutta* ) and chicken breast fillets during chilled storage, respectively.

### 3.2 Moisture Content (MC)

The ostrich flesh samples had an initial moisture level of 75%, as shown in Figure 1b. Over time, the moisture level of all the samples dropped; however, the uncoated control sample had a greater drop in moisture content than the other samples. Other researchers have reported comparable results in this area (Alizadeh Behbahani et al. 2021; Faradonbeh et al. 2024; Heydari et al. 2020). When meat is stored in the refrigerator, moisture can evaporate from its surface. This is especially true if the meat is not properly packed, allowing air to circulate around it. It has been found that edible coatings, by forming a physical barrier and preventing water vapor from migrating from the sample's surface to the outside, minimize water loss during storage (Alizadeh Behbahani et al. 2021). In other words, the presence of an active coating can help retain water by limiting the migration of water vapor from the meat to the outside.

### 3.3 Thiobarbituric Acid Reactive Substances (TBARS)

Lipid oxidation significantly influences the shelf life of food, particularly products with high fat content such as meat and meat products. Thiobarbituric acid reactive substances (TBARS) is a commonly used index to assess the extent of lipid oxidation: Higher TBARS values indicate a greater degree of fat oxidation (Mojaddar Langroodi et al. 2018). Hydroperoxides, being highly unstable compounds, do not typically contribute to the unpleasant sensory characteristics associated with lipid oxidation until they break down into secondary products. Therefore, the value of TBARS primarily reflects the presence of secondary products of lipid oxidation, particularly aldehydes (Alizadeh‐Sani et al. 2020; Majdinasab et al. 2020). Indeed, research has shown that a TBARS value exceeding 0.5 mg/kg indicates some level of oxidation, often resulting in a rancid taste. When the TBARS value surpasses 1.0 mg/kg, it suggests severe spoilage and oxidation, rendering the meat inedible (Reitznerová et al. 2017). As observed in Table 1, the TBARS values (mg/kg) of ostrich meat (OM) ranged from 0.18 to 0.19 on Day 0. Throughout the storage period, the TBARS values of all samples exhibited a consistent increase, with the highest value measured for the control group on Day 15. Notably, ostrich meat samples coated with chitosan (CS)‐based solutions showed a slower rate of increase in TBARS values during refrigerated storage. This can be attributed to the antioxidant properties and oxygen permeability barrier of chitosan, which play a key role in controlling lipid oxidation in ostrich meat (Nagarajan et al. 2021; Ojagh et al. 2010). Similar findings have been reported for other meat products in previous studies (Alizadeh Behbahani et al. 2024; Isvand et al. 2024; Tanavar et al. 2021). The antioxidant activity of chitosan stems from the presence of hydroxyl and amino groups in its structure. Its mechanism involves the primary amino groups of chitosan forming stable complexes with volatile aldehydes like malondialdehyde, which are produced during the breakdown of fats during oxidation. This interaction helps neutralize the harmful effects of lipid oxidation, thereby preserving the quality of the food product (Shahidi et al. 1999). The study of Zheng et al. (2023) showed that the application of chitosan coating on the surface of chicken breast fillets acts as a barrier against oxygen penetration and thus helps to slow down lipid oxidation.

**TABLE 1:** Changes in TBARS and TVB‐N, and PV of the ostrich meat samples during refrigerated storage (4°C) for 15 days.

	Samples	Storage time (Days)		
		0	7	15
TBARS (mg/kg)	**Control**	0.18 ± 0.01^Ac^	0.63 ± 0.13^Ab^	1.62 ± 0.10^Aa^
	**CS**	0.16 ± 0.01^Ab^	0.29 ± 0.11^Bb^	0.86 ± 0. 15^Ba^
	**FE**	0.17 ± 0.03^Ab^	0.30 ± 0.10^Bb^	0.84 ± 0.13^Ba^
	**CS + FE**	0.19 ± 0.02^Ab^	0.27 ± 0.15^Bb^	0.53 ± 0.08^Ca^
TVB‐N (mg/100 g)	**Control**	9.21 ± 0.11^Ac^	24.17 ± 0.67^Ab^	32.75 ± 0.44^Aa^
	**CS**	9.32 ± 0.24^Ac^	15.49 ± 0.45^Bb^	18.60 ± 0.12^Ba^
	**FE**	9.07 ± 0.18^Ac^	14.28 ± 0.49^Cb^	17.70 ± 0.18^Ca^
	**CS + FE**	9.43 ± 0.21^Ac^	13.35 ± 0.17^Db^	15.14 ± 0.11^Da^
PV (meq/kg)	**Control**	0.98 ± 0.07^Ac^	4.74 ± 0.23^Ab^	6.51 ± 0.39^Aa^
	**CS**	0.99 ± 0.12^Ac^	2.45 ± 0.36^Bb^	3.20 ± 0.26^BCa^
	**FE**	1.14 ± 0.14^Ac^	2.18 ± 0.17^BCb^	3.46 ± 0.23^Ba^
	**CS + FE**	1.01 ± 0.12^Ac^	1.92 ± 0.13^Cb^	2.64 ± 0.48^Ca^

*Note:* Different capital letters in each column indicate significant differences among treatments and different small letters in each row indicate significant differences in storage days (*p* < 0.05).

At the end of the storage period (Day 15), it was observed that samples coated with CS + FE exhibited lower TBARS values compared to both the control and samples coated with either CS or FE alone (as shown in Table 1). This result suggests that the coating solution containing both chitosan (CS) and 1.5% fennel extract (FE) has the most effective antioxidant capacity among the tested formulations (*p* < 0.05). Following our findings, Mojaddar Langroodi et al. (2021) stated that the addition of a combination of grape seed extract and *Origanum vulgare* essential oil to chitosan coating had a significant impact on delaying lipid oxidation in turkey meat. In addition, Isvand et al. (2024) also found that the antioxidant activity of chitosan coating is substantially enhanced by incorporating *Citrus limon* essential oil into it. Fennel extract, known for its high content of phenolic compounds such as gallic acid, kaempferol, chlorogenic acid, and protocatechuic acid, serves as a natural antioxidant and significantly delays the peroxidation of lipids (Mohamad et al. 2011). Landi et al. (2024) showed that the treatment of minced pork meat with fennel seeds can significantly reduce lipid oxidation during storage. These findings suggest that the simultaneous application of fennel extract (FE) and chitosan (CS) had an additive impact, effectively reducing lipid oxidation in ostrich meat (OM) during cold storage.

### 3.4 Total Volatile Base Nitrogen (TVB‐N)

The total volatile basic nitrogen (TVB‐N) index is a key indicator used to assess the freshness and microbiological quality of meat and meat products. During the storage of meat products, enzymatic and bacterial activities lead to the decarboxylation and deamination of proteins, resulting in the production of alkaline substances such as nitrogen, ammonia, and primary, secondary, and tertiary amines. The levels of these substances are directly correlated with product freshness, making TVB‐N a valuable indicator of quality over time (Nouri Ala and Shahbazi 2019; Sun et al. 2021). In Table 1, the TVB‐N content of treated ostrich meat samples during 15 days of storage in the refrigerator is listed. On Day 0, the TVB‐N values (mg of nitrogen/100 g of ostrich meat) ranged between 9.07 and 9.43 for both control and treated samples. These initial values were relatively low, indicating that the ostrich meat samples had appropriate quality, consistent with the low initial microbial count discussed in Section 3.6. The TVB‐N index of all treatments progressively increased throughout cold storage, with a notably faster increase observed for the control sample, reaching 24.17 on the 7th day and 32.75 on the 15th day (*p* < 0.05). However, the incorporation of FE into the CS coating significantly inhibited the formation of TVB‐N throughout the storage period. The TVB‐N value of the CS + FE coated sample was 15.14 after 15 days of storage. Considering that a content of 25 mg N per 100 g of meat and meat products is considered appropriate for TVB‐N levels (Rouhi et al. 2024), the TVB‐N values of coated samples in our study remained below this acceptable limit during the storage period, while exceeding this limit for the control sample. Similarly, Song et al. (2024) found that the addition of fennel essential oil/hydroxypropyl‐β‐cyclodextrin inclusion complex to chilled pork resulted in a significant reduction in TVB‐N values. Sun et al. (2021) also reported that the use of chitosan/sodium alginate/porous starch–fennel extract microcapsules in pork minced meat resulted in the TVB‐N value remaining below 15 even after 10 days of cold storage.

The relatively rapid increase in TVB‐N value in the control sample is primarily attributed to the breakdown of proteins and other nonprotein nitrogenous compounds, including free amino acids, ammonia, amines, and nucleotide catabolites. This breakdown occurs as a result of the activity of bacteria and endogenous enzymes during storage. In contrast, the lower values of TVB‐N observed in the treated samples can be attributed to the antibacterial and antioxidant activities of the CS + FE coatings. These activities weaken the ability of bacteria to oxidize nonprotein nitrogenous compounds and reduce the number of proteolytic bacteria present in the meat, thus slowing down the increase in TVB‐N values (Huang et al. 2020).

### 3.5 Peroxide Value (PV)

Hydroperoxides are the primary products of the lipid oxidation process, and their decomposition gives rise to secondary oxidative products such as aldehydes, ketones, and alcohols. These secondary products are responsible for the development of off‐odors and unpleasant tastes in meat products. Consequently, measuring peroxide value (PV) is a useful method for evaluating the oxidative progression in meat (Majdinasab et al. 2020). The effect of coatings on peroxide value (PV) trends of ostrich meat samples during storage at 4°C for 15 days is illustrated in Table 1. Initially, the PV (meq/kg) of the samples ranged from 0.98 to 1.14, indicating their freshness. Over time, the PV of both the control and coated samples increased, indicating oxidation of some unsaturated lipids. However, the maximum PV was observed for the control sample (6.51), which significantly differed from the values measured for samples treated with CS, FE, and CS + FE coatings (3.2, 3.46, and 2.64, respectively) on the 15th day (*p* < 0.05). This difference confirmed that the formation of hydroperoxides in the treated samples was much lower than in the control, mainly attributed to the metal‐chelating and oxygen barrier properties of chitosan‐based coatings, as well as the antioxidant activities of FE.

The rapid increase in peroxide value (PV) in uncoated meat is largely attributed to the growth of microorganisms. Microbial enzymes can facilitate the oxidation and hydrolysis of lipids, leading to an increase in PV. The extent of lipid oxidation in meat is influenced by factors such as fat content and composition, storage conditions (e.g., exposure to light, humidity, oxygen, and temperature), and the inclusion of antioxidant compounds (Alizadeh‐Sani et al. 2020). The results of the present study confirm that the developed coatings are highly effective in delaying the production of primary lipid oxidation products during refrigerated storage. These findings are consistent with previous research conducted by other researchers (Alizadeh Behbahani and Imani Fooladi 2018; Farokhzad et al. 2023).

### 3.6 Antimicrobial Analyses

#### 3.6.1 Total Viable Count (TVC)

**FIGURE 2:**
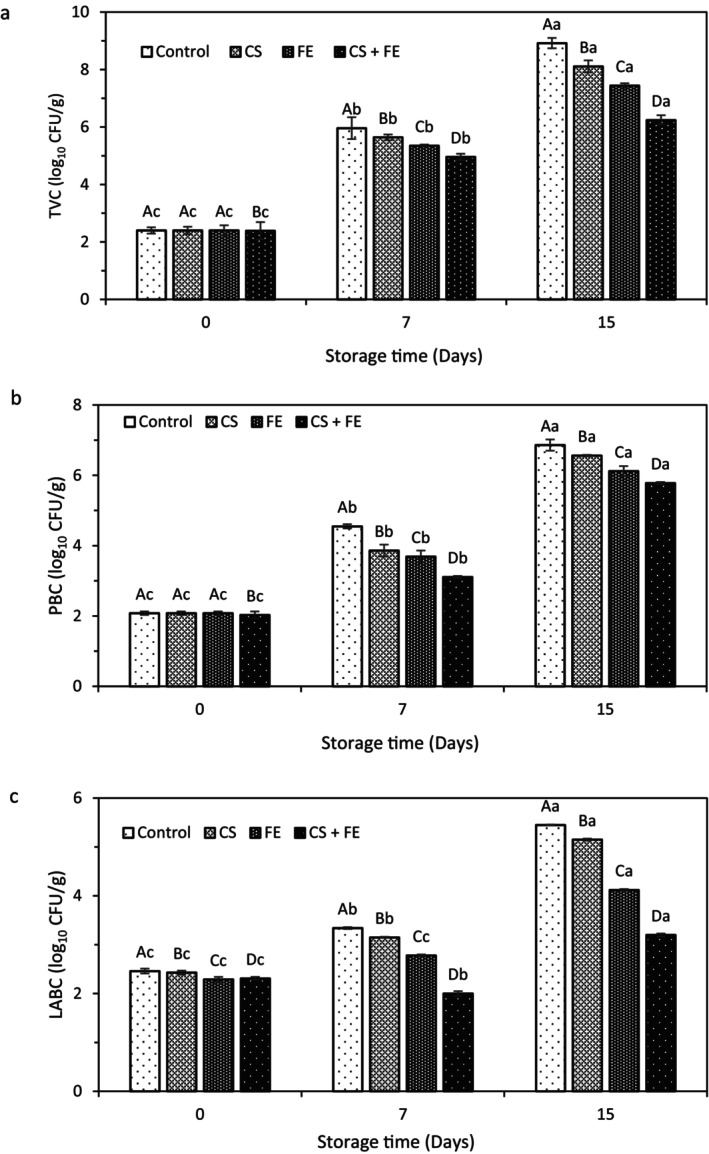
Changes in TVC (a), PVC (b), and LABC (c) of the ostrich meat samples during refrigerated storage (4°C) for 15 days. Different capital letters indicate significant differences among treatments, and different small letters indicate significant differences in storage days (*p* < 0.05).

Fresh meat is highly susceptible to microbial contamination at every stage, from production to consumption. Microbial growth and metabolism are among the leading causes of spoilage during storage. Total viable count (TVC) measurement serves as a key indicator for assessing bacterial contamination and gauging the freshness of meats. The changes in TVC of ostrich meat samples over storage time at 4°C are depicted in Figure 2a. Initially, the TVC (log_10_ CFU/g) in ostrich meat samples ranged from 2.39 to 2.40. By the 7th day of storage, the TVC in ostrich meat for all treatments remained below 6. However, by the 15th day, the TVC of the control sample and samples treated with CS, FE, and CS + FE increased to 8.92, 8.11, 7.44, and 6.24, respectively. Consistent with our findings, an increase in TVC in ostrich meat during cold storage has been reported by Heydari et al. (2020). According to the International Commission on Microbiological Specifications for Foods (ICMSF, 1986), the microbiological limit for TVC is 7. Based on this standard, at the end of the 15th day of storage, only the CS + FE coated sample remained within the permissible limit, indicating that these two compounds exhibit a clear additive effect with each other in inhibiting microbial growth.

The antimicrobial properties of chitosan coating have been documented in numerous articles (Barik et al. 2024; Chen et al. 2024). Cheng et al. (2021) found that When beef slices are chilled, the growth of microorganisms can be successfully inhibited by CS coatings in conjunction with ε‐polylysine. There are several possible explanations for chitosan's antibacterial qualities. The primary mechanism involves the presence of free amino groups on the glucosamine monomer, which gives CS a positive charge. This charge can interact with the negatively charged microbial cell membrane, causing cellular components to leak out and leading to microbial death (Kang et al. 2007). Furthermore, because of its ability to act as a barrier against the transfer of oxygen, the rate of bacterial reproduction is decreased (Ojagh et al. 2010). Fennel extract contains powerful antibacterial effects in addition to chitosan. A significant amount of Antol (about 50%–60%), which has potent antibacterial properties (Safaei‐Cherehh et al. 2020), and several phenolic compounds (flavonoids and phenolic acids) in FE can inhibit microbial growth in FE‐treated samples. Flavonoids cause disruption and inhibition in nucleic acid synthesis, cell membrane synthesis, biofilm formation, ATP synthesis, and electron transport chain. The antimicrobial mechanism of phenolic acids is that they destabilize the cytoplasmic membrane and microbial cell surfaces, and as a result, cause serious damage to various intracellular organelles and cell walls. This process involves the inhibition of intracellular enzymes and coagulation of cellular components. Additionally, when the cell wall is compromised, phenolic compounds can interact with DNA and intracellular components. As a result of internal membrane disruption, free radicals are produced, which in turn stimulate lipid oxidation and further DNA damage (Egidio et al. 2024).

#### 3.6.2 Psychrotrophic Bacteria Count (PBC)

*Pseudomonas* species and other phytotrophic bacteria are the primary culprits behind the spoilage of fresh meat and fish products stored in cold or frozen conditions. The effects of active coating on the PBC during a 15‐day storage period at 4°C are shown in Figure 2b. The data showed that the control samples' initial PBC (log_10_ CFU/g) was 2.08 and remarkably increased to more than 6 (6.86) on the final day of storage (*p* < 0.05). Although the PBC increased in all samples during storage, the ostrich meat coated with chitosan (CS) containing 1.5% FE exhibited the lowest PBC values. This suggests that this treatment had significant antimicrobial activity. The research conducted by Ghaderi et al. (2024) revealed that a gelatin/chitosan nanocomposite film incorporating *L. nobilis* essential oil nanoemulsion demonstrated significant preservative properties for ostrich meat hamburgers over 28 days of refrigerated storage. This film effectively suppressed the growth of various microorganisms, including TPC, TVC, *Salmonella*, coliforms, *S. aureus*, and *E. coli* . Also, El Bayomi et al. (2023) reported that rabbit meat treated with a combination of 1% CS and 0.2% rosemary EOs dramatically decreased the enumeration of psychrotrophic bacteria, which is consistent with the findings of the current study.

#### 3.6.3 Lactic Acid Bacteria Count (LABC)

LAB are Gram‐positive bacteria that play an important role in meat spoilage. The most species involved in this process include *Lactobacillus* spp., *Carnobacterium* spp., and *Leuconostoc* spp. (Lashkari et al. 2020). The count of LAB in control and treated ostrich meat samples stored at 4°C for 15 days is shown in Figure 2c. Initial LAB counts (log_10_ CFU/g) varied insignificantly between samples (*p* > 0.05), a gradual increase in LABC was quite evident. LAB counts in the uncoated control sample increased drastically from 2.46 on Day 0 to 5.45 after 15 days (*p* < 0.05). As expected, at the end of the storage period, the lowest LAB count was observed in the OM samples coated with CS + FE.

Based on the obtained data, it can be concluded that the reduction in LAB enumeration in the treated meat samples resulted from the effective antimicrobial action of chitosan in combination with fennel extract. Additionally, it has been proven that Gram‐positive bacteria are more sensitive to phenolic compounds than Gram‐negative bacteria. The direct contact of the constituent compounds of the extract with the phospholipid membrane of Gram‐positive bacteria leads to ion permeability and eventually the leakage of vital intracellular components or disruption of the bacterial enzyme system (Majdinasab et al. 2020).

### 3.7 Sensory Evaluation

Changes in the organoleptic characteristics of different samples of ostrich meat on Days 0, 7, and 15 of storage at 4°C are shown in Table 2. Odor, color, texture, and overall acceptability were evaluated by trained panelists, with meat samples receiving a sensory score lower than 4 considered unacceptable for consumption (Fan et al. 2008). On Day 0, all samples received high scores for color, odor, texture, and overall acceptability, indicating fresh and high‐quality meat. There was no significant difference (*p* > 0.05) in the initial sensory properties of the different samples, showing that CS coating alone or enriched with FE had no negative effect on the organoleptic characteristics of ostrich meat. However, the control sample became smelly, slimy, and exhibited color change after 7 days of refrigerated storage due to high lipid oxidation and microbial growth, rendering its sensory characteristics unacceptable. Samples treated with CS or FE retained suitable sensory scores on the 7th day, whereas only CS + FE coated samples obtained a sensory score higher than 4 even after 15 days of cold storage. These findings demonstrated that chitosan coating enriched with fennel extract can significantly retain the sensory properties of ostrich meat for more than 2 weeks. The results of the sensory evaluation are related to the analysis of the microbial and physicochemical properties, concluding that the antimicrobial, antioxidant, and oxygen barrier effects of the used coatings minimize oxidative effects, maintain quality, and extend shelf life. Similar results have been reported in previous studies, such as chitosan coating combined with an Active AgIon Absorbent Pad effectively enhancing sensory properties and prolonging the shelf life of fresh beef during storage at 5°C (Komodromos et al. 2024).

**TABLE 2:** Changes in odor, color, texture, and overall acceptability of the ostrich meat samples during refrigerated storage (4°C) for 15 days.

	Samples	Storage time (Days)		
		0	7	15
Odor	**Control**	4.93 ± 0.06^Aa^	3.60 ± 0.36^Bb^	1.43 ± 0.76^Bc^
	**CS**	4.83 ± 0.06^ABa^	4.70 ± 0.10^Aa^	2.07 ± 0. 55^Bb^
	**FE**	4.80 ± 0.10^ABa^	4.77 ± 0.15^Aa^	3.30 ± 0.44^Ab^
	**CS + FE**	4.77 ± 0.06^Ba^	4.73 ± 0.15^Aa^	4.17 ± 0.35^Ab^
Color	**Control**	4.93 ± 0.06^Aa^	3.23 ± 0.25^Bb^	1.95 ± 0.45^Cc^
	**CS**	4.83 ± 0.06^Aa^	4.50 ± 0.36^Aa^	2.93 ± 0.60^Bb^
	**FE**	4.90 ± 0.10^Aa^	4.63 ± 0.38^Aa^	3.86 ± 0.45^Ab^
	**CS + FE**	4.80 ± 0.10^Aa^	4.72 ± 0.28^Aa^	4.13 ± 0.21^Ab^
Texture	**Control**	4.87 ± 0.06^Aa^	3.96 ± 0.15^Cb^	2.17 ± 0.21^Cc^
	**CS**	4.93 ± 0.12^Aa^	4.47 ± 0.16^Bb^	3.57 ± 0.25^Bc^
	**FE**	4.97 ± 0.06^Aa^	4.37 ± 0.35^ABb^	3.73 ± 0.30A^Bc^
	**CS + FE**	4.80 ± 0.10^Aa^	4.83 ± 0.11^Aa^	4.11 ± 0.10^Ab^
Overall acceptability	**Control**	4.90 ± 0.10^Aa^	3.80 ± 0.40^Bb^	1.16 ± 0.15^Cc^
	**CS**	4.80 ± 0.10^Aa^	4.83 ± 0.11^Aa^	2.34 ± 0.35^BCb^
	**FE**	4.83 ± 0.12^Aa^	4.73 ± 0.38^Aa^	3.80 ± 0.36^Ab^
	**CS + FE**	4.83 ± 0.15^Aa^	4.87 ± 0.12^Aa^	4.34 ± 0.35^Ab^

*Note:* Different capital letters in each column indicate significant differences among treatments and different small letters in each row indicate significant differences in storage days (*P* < 0.05).

## 4 Conclusion

The results of the present study showed that the treatment of fresh ostrich meat using FE‐enriched CS coating could effectively control pH and MC changes and limit the increase in TBARS, TVB‐N, and PV content over 15 days of storage at 4°C. Both CS and FE exhibited antimicrobial properties against mesophilic, psychrotrophic, and lactic acid bacteria, with a significant additive effect when combined. The CS and FE coating treatment not only avoided any adverse effects on the odor, color, texture, and overall acceptability of ostrich meat samples but also preserved their sensory characteristics throughout the storage period. Based on these findings, the chitosan coating enriched with fennel extract offers a natural preservative solution that can be used to maintain quality and extend the shelf life of meat and meat products, with potential applications in the meat industry. However, further research is needed to evaluate the effect of the encapsulation process and the use of other natural extracts in combination with chitosan coating on the physicochemical, microbial (especially specific species and pathogens), sensory, nutritional properties as well as the shelf life of fresh ostrich meat.

## Author Contributions

**Mahdieh Salari:** conceptualization (equal), project administration (equal), writing – original draft (equal). **Motahareh Yazdany:** investigation (equal), methodology (equal).

## Ethics Statement

This study does not involve any human or animal experiments.

## Consent

Written informed consent was obtained from all participants in the study.

## Conflicts of Interest

The authors declare no conflicts of interest.

## Data Availability

All the data used is shown in this article.
